# Targeting glial activation to mitigate mirror‐image and extraterritorial neuropathic pain in a CCI model of neuropathic pain in male rats

**DOI:** 10.14814/phy2.70318

**Published:** 2025-04-23

**Authors:** Mohammad‐Shafi Mojadadi, Bahareh Amin, Hossein Zeinali, Samad Nazemi

**Affiliations:** ^1^ Department of Immunology, School of Medicine Sabzevar University of Medical Sciences Sabzevar Iran; ^2^ Department of Physiology and Pharmacology, School of Medicine Sabzevar University of Medical Sciences Sabzevar Iran; ^3^ Department of Physiology and Pharmacology, School of Medicine Qom University of Medical Sciences Qom Iran; ^4^ Department of Physiology and Pharmacology, Cellular and Molecular Research Center, School of Medicine Sabzevar University of Medical Sciences Sabzevar Iran

**Keywords:** CCI model, minocycline, mirror‐image pain, neuropathic pain, pentoxifylline

## Abstract

Neuropathic pain (NP) arises from nerve damage or compression and often extends to the contralateral side of the body (mirror‐image pain, MP) and adjacent non‐injured areas (extraterritorial pain). This study investigates whether altered sensitivity in these contralateral and peripheral regions is mediated by glial cells, using the chronic constriction injury (CCI) model of NP. Thirty‐two male Wistar rats were randomly assigned into four groups (8/group): sham, CCI + vehicle, CCI + minocycline (MIN;10 mg/kg), and CCI + pentoxifylline (PTX;8 mg/kg). The CCI model was employed for NP induction. MIN and PTX were administered intraperitoneally from postoperative days (POD)4 to POD14, once daily. Pain responses were assessed on POD0, 2, 6, 10, and14 using Hargreaves, von Frey, and Tail‐flick tests. Western blot analysis was performed on POD14 to measure Iba1 and GFAP protein expression in the spinal cord hemispheres. Results revealed that post‐injury treatment with MIN and PTX significantly reduced contralateral thermal hyperalgesia, mechanical allodynia, and tail‐flick responses. Correspondingly, the contralateral spinal cord exhibited significantly decreased GFAP and Iba1 protein expression compared to the CCI + vehicle treated group. These findings suggest that post‐injury glial cell inhibition effectively mitigates neuropathic pain and prevents the development of MP and extraterritorial pain. This highlights the potential for clinical applications targeting glial cells to manage NP even after nerve injury.

## 1 INTRODUCTION

Neuropathic pain is a complex and often debilitating condition, arising as a direct outcome of a disease or lesion affecting the somatosensory system (Cao et al., 2024; Finnerup et al., 2021). This type of pain is a challenging chronic condition and continues to be a significant problem. Effective solutions for pain relief remain an unmet global need for every patient (Moisset, 2024; Sadegh et al., 2024). Altered sensitivity in contralateral healthy structures or regions not directly innervated by the injured nerve has been demonstrated in various experimental pain models and clinical settings, a phenomenon called “extraterritorial” or “mirror‐image” pain (MP) (Cheng et al., 2014; Drinovac Vlah & Bach‐Rojecky, 2024; Kambiz et al., 2015; Malan et al., 2000). Recently, the investigation of mirror‐image neuropathic pain has been facilitated by developing various animal pain models with unilateral nerve injury or inflammation (Arguis et al., 2008; Luz et al., 2023; Yang et al., 2023). The neural mechanism underlying MP is controversial. Evidence suggests that increased spinal cord levels of substance P, NMDA receptors, and dynorphin contribute to MP development after nerve injury (Fang et al., 2023; Senba et al., 2012).

The role of glial cells in MP is intriguing and has garnered growing interest. Astrocytes and microglial cells exhibit heightened activity in the contralateral dorsal spinal cord after unilateral nerve injury, as indicated by elevated expression of glial activation markers such as OX‐42 and glial fibrillary acidic protein (GFAP) (Gao et al., 2010). While glial activation occurs rapidly in the ipsilateral spinal cord hemisphere, activation in the contralateral hemisphere presents a temporal delay (Koltzenburg et al., 1999). Targeting glial cells as a therapeutic approach has shown promise in neurodegenerative disorders and neuropathic pain models (Magni et al., 2024). For instance, preemptive administration of minocycline (MIN), a second‐generation tetracycline, effectively alleviates NP symptoms. MIN suppresses microglial activation and proliferation, reducing proinflammatory cytokines like TNF‐α, IL‐1, IL‐6, and IL‐8 (Colovic & Caccia, 2003; Guasti et al., 2009). Similarly, pentoxifylline (PTX), a nonspecific cytokine and phosphodiesterase inhibitor, has demonstrated efficacy in reducing cytokine synthesis and mitigating NP symptoms (Lundblad et al., 1995; Zanjani et al., 2006, 2010).

However, the impact of these glia‐targeting drugs on contralateral and extraterritorial pain following injury remains underexplored. This study employs a chronic constriction injury (CCI) model to evaluate the effects of post‐injury administration of MIN and PTX on MP and extraterritorial pain in rats. By targeting glial activation and cytokine release, this research aims to elucidate whether these interventions could offer effective relief from contralateral and widespread pain symptoms associated with neuropathy.

## 2 MATERIALS AND METHODS

### 2.1 Ethics and drugs

All experimental procedures adhered to the Guidelines on Ethical Standards for Investigations of Experimental Pain in Animals and were approved by the Ethics Committee of Sabzevar University of Medical Sciences (Approval ID: IR.MEDSAB.REC.1393.009). The study was conducted in accordance with NIH guidelines. Reagents were sourced from Sigma‐Aldrich (Sigma‐Aldrich Co. LLC, USA).

### 2.2 Animals

Male Wistar rats (weighing 230 ± 20 g and 8 weeks old) were procured from the Pasteur Research Center in Karaj, Iran. Male rats were selected to avoid potential hormonal variations seen in female rats, which could influence pain responses and glial activation. Animals were housed under controlled environmental conditions: temperature (24 ± 2°C), humidity (50 ± 5%), and a 12‐h light/dark cycle. Food and water were provided ad libitum. The animals were fed a standard rodent diet (pelleted chow, Pars Animal Feed, Iran).

### 2.3 Induction of neuropathic pain using the CCI model

Neuropathic pain was induced using the chronic constriction injury (CCI) model described by Bennett and Xie (1988). Rats were anesthetized with sodium pentobarbital (60 mg/kg, intraperitoneally). The biceps femoris and gluteus superficialis muscles were separated to expose the right sciatic nerve. Four loose ligatures (4–0 catgut) were tied around the sciatic nerve just proximal to its trifurcation, ensuring the ligatures were tight enough to elicit a brief muscle twitch in the hind limb (Bennett & Xie, 1988). The wound was then closed by suturing the muscle and skin. For sham surgery, the sciatic nerve was exposed without applying ligatures. Post‐surgery, animals were allowed to recover in their home cages. No prophylactic antibiotics were administered, and aseptic surgical techniques were strictly followed to minimize infection risk (Nazemi et al., 2021).

### 2.4 Experimental protocol

Thirty‐two male rats were randomly allocated into four groups (8 rats per group):

1. Sham group: Underwent sham surgery and received no treatment.
2. CCI‐vehicle treated group: Subjected to CCI and treated with normal saline (vehicle).
3. MIN group: Underwent CCI and received minocycline (10 mg/kg), Sigma‐Aldrich (Catalog No. M9511).
4. PTX group: Underwent CCI and received pentoxifylline (8 mg/kg), Sigma‐Aldrich (Catalog No. P1784).

These dosages were likely chosen based on prior researches demonstrating effective neuroprotection and inflammation reduction in CCI models without excessive toxicity (Nazemi et al., 2012).

Drugs were administered intraperitoneally from postoperative day (POD) 4 to POD 14, once daily. Behavioral tests were conducted in a blinded manner on POD 0, 2, 6, 10, and 14. Efforts were made to minimize animal discomfort throughout the study.

### 2.5 Hargreaves test for thermal hyperalgesia

Thermal hyperalgesia was evaluated using the Hargreaves test, which measures paw withdrawal latency. Rats were placed in individual plastic enclosures on a heat‐tempered glass floor (Plantar Test, Ugo Basile, Italy) and habituated for 15 min. A radiant heat source (50 W halogen bulb) was focused on the plantar surface of both hind paws. Each paw was tested three times at 5‐min intervals, and the average withdrawal latency was recorded. A cutoff time of 33 s was used to prevent tissue damage if no withdrawal occurred (Nazemi et al., 2015).

### 2.6 Tactile sensitivity test (von Frey test)

Mechanical allodynia was assessed using calibrated von Frey monofilaments (Stoelting, Wood Dale, IL). Rats were acclimated in transparent enclosures with a wire mesh floor for 15 min. Filaments were applied perpendicularly to the plantar surface of both hind paws with enough pressure to cause slight buckling. Each stimulus lasted 5 s, and a withdrawal or flinch response was recorded as positive. The force required for a 50% likelihood of withdrawal was calculated using the up‐down method. A cutoff force of 60 g was applied to avoid tissue damage (Chaplan et al., 1994; Nazemi et al., 2024).

### 2.7 Thermal nociceptive threshold (tail‐flick test)

Thermal nociceptive thresholds were determined using an automated tail‐flick analgesia meter (Ugo Basile, Comerio, Italy). A focused beam of light was directed at the dorsal surface of the tail, approximately 2.5 cm from the tip. The time between stimulus onset and tail flick was recorded. Baseline latencies were adjusted between 8 and 11 s, with a 20‐s cutoff to avoid tissue damage (Nishiyama & Hanaoka, 2001).

### 2.8 Western blot

On POD 14, rats were euthanized and the lumbar spinal cord (L5–L6) was dissected. Euthanasia was performed by inducing deep anesthesia with isoflurane (5% in oxygen) via inhalation, followed by decapitation using a rodent guillotine. Samples from ipsilateral and contralateral sides were homogenized in RIPA buffer, and protein concentrations were quantified using the Bradford method. Sixty micrograms of protein per sample were resolved by SDS‐PAGE and transferred to PVDF membranes. Membranes were blocked and incubated with primary antibodies against GFAP protein as an astroglia marker (Monoclonal, Clone S.880.0, Catalog No. MA5‐15086) and Iba1 protein as a microglial marker (Polyclonal, Wako Chemicals, Catalog No. 019‐19741). After washing, membranes were incubated with anti‐goat (1∶5000; cat. sc‐2020, Santa Cruz Biotechnology) secondary antibodies, and immunoreactivity was visualized using an enhanced chemiluminescence (ECL) detection system (cat. RPN2106, GE Healthcare). β‐actin served as the loading control, and band intensities were analyzed with ImageJ software (V1.41; NIH, Bethesda, MD, USA) after background subtraction. Protein expression levels were normalized to β‐actin and expressed as relative fold changes compared to the sham group.

### 2.9 Statistical analysis

Data were analyzed using IBM SPSS software version 16 (IBM Corp., Armonk, NY, USA). Results are presented as mean ± standard deviation (SD). The normality of the data was assessed using the Shapiro–Wilk test. Comparisons among groups were performed using one‐way analysis of variance (ANOVA). Post hoc analysis was conducted with Tukey's test to identify significant differences between groups. A significance level of *p* < 0.05 was considered statistically significant.

## 3 RESULTS

### 3.1 Post‐injury treatment with minocycline and pentoxifylline reduced contralateral thermal hyperalgesia (Hargreaves test)

The effect of post‐injury administration of minocycline (MIN) and pentoxifylline (PTX) on thermal hyperalgesia in the ipsilateral and contralateral hind paws was assessed using the Hargreaves test. One‐way ANOVA indicated significant changes in paw withdrawal latency (PWL) in both the ipsilateral (*F*_3,23_ = 3.34, *p* < 0.005) and contralateral hind paws (*F*_3,23_ = 2.66, *p* < 0.01).

**FIGURE 1:**
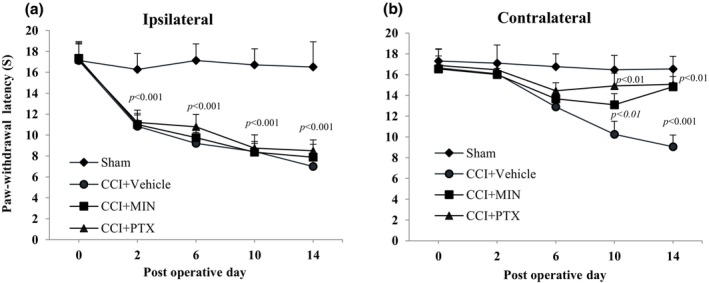
Effect of intraperitoneal administration of vehicle (V), minocycline (MIN; 10 mg/kg), and pentoxifylline (PTX; 8 mg/kg) from postoperative day (POD) 4 to 14 (once daily) on thermal hyperalgesia in CCI rats. Graphs show paw withdrawal latency (PWL) in the (a) ipsilateral and (b) contralateral hind paws. Statistical analysis was performed using mixed‐model ANOVA followed by the Bonferroni multiple comparison test (*n* = 8). Data are presented as mean ± SD. CCI, chronic constriction injury; MIN, minocycline; PTX, pentoxifylline; PWL, paw withdrawal latency.

Tukey's post hoc analysis revealed a significant reduction in PWL in the ipsilateral hind paws of the chronic constriction injury (CCI) group compared to the Sham group from postoperative day (POD) 2 to POD 14 (*p* < 0.005). A similar decrease was observed in the contralateral hind paws from POD 10 to POD 14 (*p* < 0.05), suggesting bilateral neuropathic pain induction. Post‐injury treatment with MIN and PTX significantly attenuated thermal hyperalgesia in the contralateral hind paw, as indicated by increased PWL from POD 10 onwards compared to the CCI‐vehicle treated group (*p* < 0.01). However, neither MIN nor PTX significantly influenced PWL in the ipsilateral hind paw (*p* > 0.05) (Figure 1a,b).

### 3.2 Post‐injury treatment with minocycline and pentoxifylline reduced contralateral mechanical allodynia (von Frey test)

The von Frey test was used to evaluate mechanical allodynia in the ipsilateral and contralateral hind paws following post‐injury administration of MIN and PTX. One‐way ANOVA revealed significant changes in paw withdrawal threshold (PWT) in both the ipsilateral (*F*_3,23_ = 2.94, *p* < 0.005) and contralateral hind paws (*F*_3,23_ = 2.76, *p* < 0.05).

**FIGURE 2:**
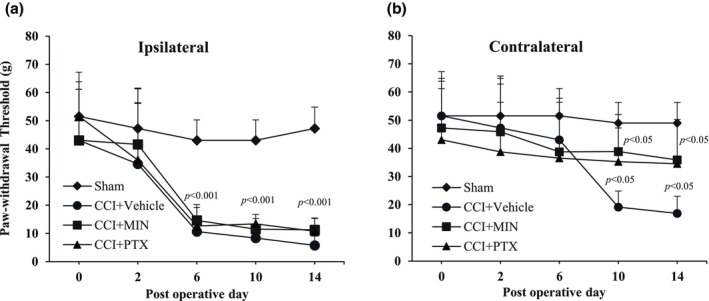
Effect of intraperitoneal administration of vehicle (V), minocycline (MIN; 10 mg/kg), and pentoxifylline (PTX; 8 mg/kg) from POD 4 to 14 (once daily) on mechanical allodynia in CCI rats. Graphs show paw withdrawal threshold (PWT) in the (a) ipsilateral and (b) contralateral hind paws. Statistical analysis was performed using mixed‐model ANOVA followed by the Bonferroni multiple comparison test (*n* = 8). Data are presented as mean ± SD. CCI, chronic constriction injury; MIN, minocycline; PWT, paw withdrawal threshold; PTX, pentoxifylline.

Tukey's post hoc analysis showed a marked reduction in PWT in the ipsilateral hind paws of the CCI‐vehicle treated group from POD 6 to POD 14 (*p* < 0.005) and in the contralateral hind paws from POD 10 to POD 14 (*p* < 0.01) compared to the Sham group. This indicates the bilateral development of neuropathic pain. Treatment with MIN or PTX significantly alleviated mechanical allodynia in the contralateral hind paws, evidenced by increased PWT from POD 10 onwards (*p* < 0.01). However, no significant effects were observed in the ipsilateral hind paws (*p* > 0.05) (Figure 2a,b).

### 3.3 Post‐injury treatment with minocycline and pentoxifylline reduced extraterritorial pain (tail‐flick test)

To evaluate the effects of MIN and PTX on extraterritorial pain, the tail‐flick test was performed. One‐way ANOVA indicated significant changes in tail‐flick latency (TFL) (*F*_3,23_ = 3.46, *p* < 0.01).

**FIGURE 3:**
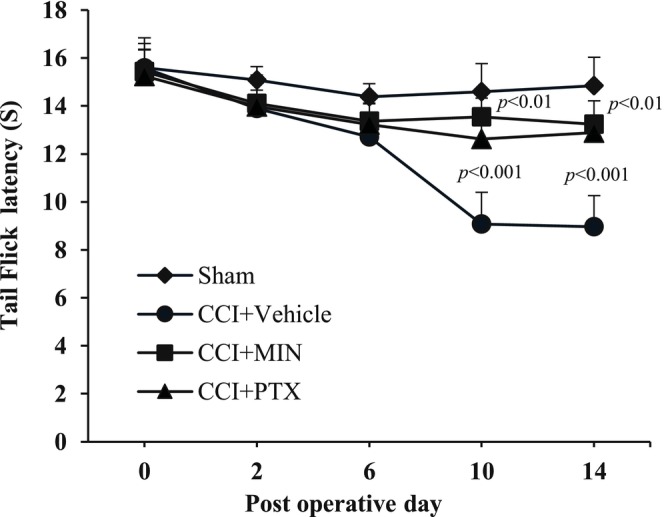
Effect of intraperitoneal administration of vehicle (V), minocycline (MIN; 10 mg/kg), and pentoxifylline (PTX; 8 mg/kg) from POD 4 to 14 (once daily) on tail‐flick latency (TFL) in CCI rats. Graphs depict TFL measurements as an indicator of extraterritorial pain. Statistical analysis was performed using mixed‐model ANOVA followed by the Bonferroni multiple comparison test (*n* = 8). Data are presented as mean ± SD. CCI, chronic constriction injury; MIN, minocycline; PTX, pentoxifylline; TFL, tail‐flick latency.

Tukey's post hoc analysis revealed that the CCI‐vehicle treated group exhibited a significant reduction in TFL from POD 10 to POD 14 compared to the Sham group (*p* < 0.005), indicating the presence of extraterritorial pain. Post‐injury treatment with MIN or PTX significantly increased TFL from POD 10 onwards compared to untreated CCI animals (*p* < 0.01). This suggests that both drugs effectively mitigate extraterritorial pain (Figure 3).

### 3.4 Minocycline and pentoxifylline treatment inhibits GFAP and Iba‐1 expression

The effect of MIN and PTX on glial activation was analyzed by measuring the expression of GFAP (astrocytic marker) and Iba1 (microglial marker) in the lumbar spinal cord via Western blot. The results revealed significant changes in GFAP and Iba1 expression in both the ipsilateral (*F*_3,23_ = 4.24, *p* < 0.05 for GFAP; *F*_3,23_ = 5.67, *p* < 0.05 for Iba1) and contralateral hemispheres (*F*_3,23_ = 2.68, *p* < 0.05 for GFAP; *F*_3,23_ = 3.16, *p* < 0.05 for Iba1).

In the CCI‐vehicle treated group, both GFAP and Iba1 levels were significantly elevated in the ipsilateral and contralateral hemispheres compared to the Sham group (*p* < 0.01), indicating glial activation due to nerve injury. However, post‐injury treatment with MIN and PTX significantly reduced GFAP and Iba1 expression in the contralateral hemisphere (*p* < 0.05), suggesting effective inhibition of glial activation. No significant reduction in GFAP or Iba1 expression was observed in the ipsilateral hemisphere following MIN or PTX treatment (*p* > 0.05).

**FIGURE 4:**
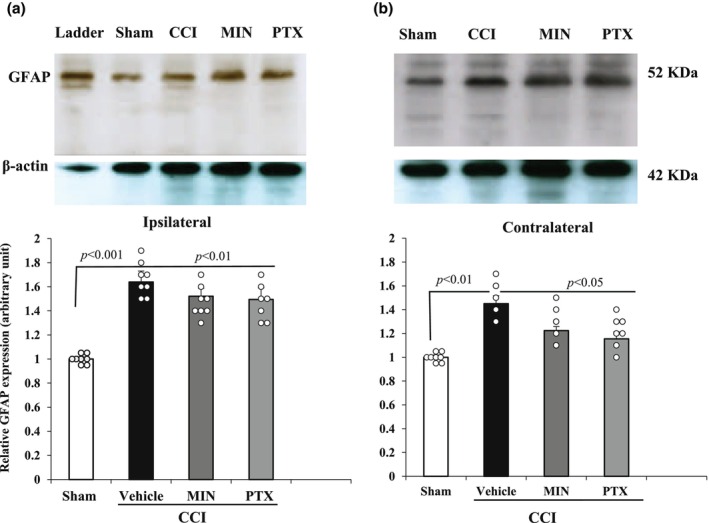
Effect of intraperitoneal administration of vehicle (V), minocycline (MIN; 10 mg/kg), and pentoxifylline (PTX; 8 mg/kg) from POD 4 to 14 (once daily) on GFAP expression in the ipsilateral and contralateral hemispheres of the lumbar spinal cord in CCI rats. Statistical analysis was conducted using one‐way ANOVA followed by Tukey's post hoc test (*n* = 8). Data are presented as mean ± SD. CCI, chronic constriction injury; GFAP, glial fibrillary acidic protein; MIN, minocycline; PTX, pentoxifylline.

**FIGURE 5:**
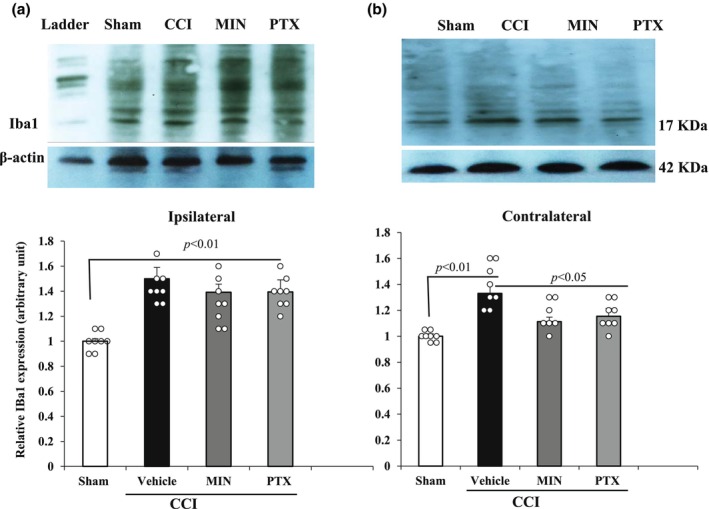
Effect of intraperitoneal administration of vehicle (V), minocycline (MIN; 10 mg/kg), and pentoxifylline (PTX; 8 mg/kg) from POD 4 to 14 (once daily) on Iba1 expression in the ipsilateral and contralateral hemispheres of the lumbar spinal cord in CCI rats. Statistical analysis was conducted using one‐way ANOVA followed by Tukey's post hoc test (*n* = 8). Data are presented as mean ± SD. CCI, chronic constriction injury; Iba1, ionized calcium‐binding adaptor molecule 1; MIN, minocycline; PTX, pentoxifylline.

These findings indicate that MIN and PTX effectively suppress glial activation in the contralateral spinal cord, which may play a role in mitigating mirror‐image and extraterritorial pain, while their effects on the ipsilateral hemisphere remain limited (Figures 4 and 5).

## 4 DISCUSSION

This study investigated the effects of post‐injury administration of minocycline (MIN) and pentoxifylline (PTX) on neuropathic pain using the chronic constriction injury (CCI) model in rats. The findings provide compelling evidence that both drugs mitigate contralateral thermal hyperalgesia, mechanical allodynia, and extraterritorial pain, primarily through the inhibition of glial activation. Importantly, the results highlight the potential of targeting glial cells to alleviate neuropathic pain beyond the site of injury, including mirror‐image and extraterritorial pain phenomena. All CCI rats showed marked guarding of the injured hind paw, none of the animals exhibited self‐mutilation behaviors or signs of severe distress, and all maintained normal grooming and eating behaviors throughout the experiment. Since no behavioral abnormalities were observed in the sham group, the bilateral symptoms in the CCI‐vehicle treated group must be attributed to unilateral sciatic nerve ligation.

The behavioral results showed a rapid, significant increase in thermal hyperalgesia and mechanical allodynia in the ipsilateral hind paw starting on day two post‐surgery and lasting until day 14, compared to the sham group. The contralateral hind paw displayed a delayed response, with increased sensitivity to thermal and mechanical stimuli starting on postoperative day (POD) 6 and continuing to increase until POD 14 (Figures 1 and 2). Previous studies also report bilateral allodynia in this neuropathic pain model (Attal et al., 2023; Benison et al., 2011; Finnerup et al., 2021). Vissers et al. (2003) found that CCI reduces bilateral responsiveness in the formalin test (Vissers et al., 2003). Additionally, unilateral sciatic nerve injury has been shown to induce bilateral elevation of IL‐6 and IL‐6R mRNA levels in the L4–L5 dorsal root ganglion (DRG), with these effects propagating to the cervical DRG, suggesting a systemic neuroinflammatory response (Brazda et al., 2013).

However, some studies report no contralateral effects after nerve injury (Clark et al., 2007). This discrepancy could be due to methodological differences. Some researchers use difference scores to compare stimulus‐evoked responses between the ligated and unoperated paws, which may overlook contralateral changes (Bennett & Xie, 1988). Variations in neuropathic pain models and behavioral tests could also explain the differences in findings.

Several mechanisms have been proposed to explain mirror‐image pain (MP), with central sensitization playing a key role. This sensitization facilitates pain transmission across neural connections between both sides of the central nervous system (Drinovac Vlah & Bach‐Rojecky, 2024). Some theories emphasize neural mechanisms, while others highlight immune responses, particularly the activation of microglia and astrocytes in the central nervous system (CNS) following peripheral nerve injury (DeLeo et al., 2004; Schreiber et al., 2008). During neuroinflammation, glial cells release mediators that can spread pain beyond the initial injury site, contributing to extraterritorial pain (Ji et al., 2018). Activated glial cells release pro‐inflammatory cytokines that enhance dorsal horn neuron sensitivity, leading to widespread pain (Andersen et al., 2014; Nazemi et al., 2025). Cytokines may spread through cerebrospinal fluid or indirectly activate contralateral glial cells, increasing dorsal neuron excitability (Ledeboer et al., 2005).

Preventing or reversing glial activation with inhibitors such as minocycline (MIN) and pentoxifylline (PTX) can alleviate neuropathic pain, especially when administered preemptively (Mika et al., 2009). However, these treatments may not reverse established hyperalgesia or allodynia (Mika et al., 2007).

This study demonstrated that unilateral chronic constriction injury (CCI) of the sciatic nerve induced neuropathic pain in the rats' hind paws and tail. While ipsilateral symptoms appeared immediately after injury, contralateral and extraterritorial changes were delayed, emerging around 6 days post‐injury.

In addition, post‐injury administration of MIN and PTX reduced contralateral thermal hyperalgesia and mechanical allodynia and decreased glial activation in the contralateral spinal cord, as evidenced by reduced Iba1 and GFAP expression. However, these doses of MIN and PTX did not alleviate ipsilateral neuropathic pain, consistent with previous findings (Nazemi et al., 2015). The delayed development of contralateral pain suggests that MIN and PTX, administered from POD 10 to 14, successfully prevented glial cell activation in the contralateral dorsal horn, possibly by reducing inflammation. Based on previous studies, we hypothesize that these drugs may modulate intracellular signaling pathways involved in glial activation. However, further investigation is required to confirm this mechanism.

The results of this study have important implications for clinical practice. Current treatments for neuropathic pain largely focus on peripheral injury sites and symptomatic management, with limited success in addressing mirror‐image or extraterritorial pain.

(Sun et al., 2023). The demonstrated efficacy of MIN and PTX in inhibiting glial activation and alleviating contralateral pain suggests that these drugs, or similar glia‐targeted therapies, could be incorporated into post‐injury treatment protocols. Importantly, these drugs exert their effects even when administered days after the injury, making them viable options for clinical intervention following delayed diagnosis (Tikka et al., 2001).

### 4.1 Limitations and future directions

While the findings are promising, this study has some limitations. First, the focus was on a single animal model (CCI), which may not capture the full spectrum of neuropathic pain mechanisms observed in humans. Second, the ipsilateral effects of MIN and PTX were minimal, raising questions about their differential action in the two hemispheres. Further research is needed to explore the underlying mechanisms driving this discrepancy. Additionally, future studies should examine the long‐term effects of these drugs and their potential to prevent chronic pain syndromes.

## 5 CONCLUSION

This study demonstrates that post‐injury treatment with minocycline and pentoxifylline effectively mitigates contralateral neuropathic pain and extraterritorial pain by suppressing glial activation in the contralateral spinal cord. These findings underscore the importance of glial cells in the propagation of neuropathic pain and highlight the potential of glial inhibitors as therapeutic agents for managing complex pain syndromes, including mirror‐image pain. Further research is warranted to translate these findings into clinical applications and develop more targeted interventions for neuropathic pain.

## ACKNOWLEDGEMENTS

During the preparation of this work, the authors used ChatGPT4 for language refinement. The final content was rigorously reviewed and approved by all authors, who assume full responsibility for its accuracy and integrity.

## FUNDING INFORMATION

The authors are grateful to the deputy of research and technology of Sabzevar University of Medical Sciences for supporting this research (Grant number: 92033).

## CONFLICT OF INTEREST STATEMENT

The authors declare no competing interests.

## Data Availability

The data of this study are available upon reasonable request from the corresponding author.
